# “It’s Embedded in What We Do for Every Child”: A Qualitative Exploration of Early Childhood Educators’ Perspectives on Supporting Children’s Social and Emotional Learning

**DOI:** 10.3390/ijerph18041530

**Published:** 2021-02-05

**Authors:** Claire Blewitt, Amanda O’Connor, Heather Morris, Andrea Nolan, Aya Mousa, Rachael Green, Amalia Ifanti, Kylie Jackson, Helen Skouteris

**Affiliations:** 1 Health and Social Care Unit, School of Public Health and Preventive Medicine, Monash University, 553 St Kilda Road, Melbourne, VIC 3004, Australia; claire.blewitt@monash.edu (C.B.); mandy.oconnor@monash.edu (A.O.); heather.morris@monash.edu (H.M.); rachael.green1@monash.edu (R.G.); 2 School of Education, Faculty of Arts and Education, Deakin University, 1 Gheringhap Street, Geelong, VIC 3220, Australia; a.nolan@deakin.edu.au; 3 Monash Centre for Health Research and Implementation, School of Public Health and Preventive Medicine, Monash University, Level 1, 42-51 Kanooka Grove, Clayton, VIC 3168, Australia; aya.mousa@monash.edu; 4 Department of Educational Sciences and Early Childhood Education, University of Patras, 26504 Patras, Greece; ifanti@upatras.gr; 5 Bestchance Child Family Care, 583 Ferntree Gully Road, Glen Waverley, VIC 3150, Australia; KJackson@bestchance.org.au; 6 Warwick Business School, Warwick University, Coventry CV4 7AL, UK

**Keywords:** early childhood, kindergarten, preschool, social and emotional learning, teacher-child interactions, qualitative research

## Abstract

Early childhood educators play an important role in supporting children’s social and emotional development. While a growing body of research has examined the impact of curriculum-based social and emotional learning (SEL) programs on child outcomes, the approaches educators use to strengthen children’s social and emotional functioning through their everyday practices are less defined. This study explored Australian early childhood educators’ perspectives on children’s social and emotional development, the approaches educators use to encourage children’s social and emotional skills, the enablers and barriers to SEL within the preschool environment, and the additional support needed. Thirty Early Childhood Education and Care professionals participated in semi-structured interviews and focus group discussions. Findings suggest children’s social–emotional development is at the forefront of educator planning, practice, and reflection. Participants described utilising various approaches to support children’s social and emotional skills, embedded within interactions and relationships with children and families. Specifically, strategies could be grouped into four broad categories: a nurturing and responsive educator–child relationship; supporting SEL through everyday interactions and practice; utilising the physical environment to encourage SEL; and working in partnership with caregivers. There was, however, inconsistency in the variety and type of approaches identified. Time constraints, group size, educator confidence and capability, high staff turnover, and limited guidance regarding high-quality social and emotional pedagogy were identified as key barriers. Participants sought practical strategies that could be embedded into daily practice to build upon current knowledge.

## 1. Introduction

Early childhood presents a unique window for social and emotional skill development. Social and emotional competence in young children has been described as an emerging ability to establish secure relationships with both adults and peers, experience, regulate and express emotions, explore the environment, and learn. This development occurs within the context of family, community and culture [1]. Frameworks of social–emotional functioning in early childhood grapple with the rapid growth that takes place during this period and the overlap between various skills and behaviours. As such, scholars often suggest domains of development and discrete skills that sit within each [2,3]. Drawing on a review of social–emotional domains most often captured in theoretical models, Halle and Darling-Churchill [4] offer social competence, emotional competence, self-regulation, and behaviour problems as central to understanding and assessing child development. Executive functioning is increasingly included as a distinct but related dimension, referring to the cognitive processes which enable children to organise their thinking and behaviour, facilitating self-regulation and learning.

This is also a period when social and emotional difficulties can first emerge. Australian research suggests between 13% and 19% of children aged 1.5 to 3 years display clinically significant difficulties with social, emotional or behavioural functioning, increasing to almost 20% by six years of age; however, only 16% of these access support from specialist mental health services [5]. Increasing understanding of the epidemiology of mental health problems indicates a robust link between social, emotional, and behavioural difficulties in childhood and later mental health problems [6,7], in addition to other maladaptive outcomes including obesity, diabetes and heart disease, lower rates of tertiary education, and reduced vocational opportunities [7,8,9,10].

The social and emotional skills that provide a foundation for ongoing learning and wellbeing are largely shaped by a child’s early relationships and care experiences [11,12]. Socioecological perspectives of child development [13] offer a framework to consider the multiple relationships and environments in which child development occurs. While family is recognised as the first and foremost influence on children’s wellbeing, other individuals, such as early childhood educators, can play an important role in supporting healthy development. Many children access Early Childhood Education and Care (ECEC) in the formative years before formal schooling [14], offering a pathway to reach children at their point of need. High-quality ECEC includes both structural and process elements. According to the Organisation for Economic Co-operation and Development, this encompasses a stimulating environment, high-quality pedagogy and teacher–child interactions, highly qualified educators, and positive working conditions [15]. Together, these factors can strengthen children’s social, emotional, and cognitive functioning and subsequent school readiness, with research suggesting improvement in short- and long-term learning, health, and vocational outcomes [16,17,18,19]. This can be especially important for children showing signs of early social and emotional difficulty, and children exposed to other individual or familial risk factors, such as socioeconomic disadvantage [20,21,22].

Knowledge of the importance of high-quality early learning experiences has seen children’s social and emotional skills prioritised in learning policy and curricula across countries, including Australia [23], the United States [24], England [25,26], and Singapore [27]. Social and emotional learning (SEL) describes the process by which children acquire and apply knowledge, skills, and attitudes relating to self-awareness, social awareness, self-management, relationship skills, and responsible decision making [28]. Research evidence shows early childhood educators can strengthen these emerging social and emotional competencies that promote future learning, health, and wellbeing in a similar way that they foster language and early literacy skills; through explicit instruction, teacher practices, integration within broader early learning pedagogy, and systemic integration across early childhood classrooms, settings, families, and communities [29,30,31]. Subsequently, there is increasing availability of SEL programming designed for ECEC to support educators to foster children’s social and emotional health [29,30,31,32,33,34].

Teachers’ perspectives on SEL can influence adoption and sustainability and may moderate impact child outcomes [35,36]. These have been investigated by examining the social validity of SEL programming [37,38,39,40], and exploration of educators’ views relating to classroom-based SEL programs [41]. In a recent qualitative study, early childhood teachers (pre-kindergarten to Grade 3) in urban classrooms in the United States contended that they, with parents, were responsible for supporting children’s social and emotional competences. Educators wanted SEL curricula but did not want scripted programs as these placed restrictions which some perceived to limit their ability to effectively implement interventions. They also highlighted that SEL programming should reflect school and classroom culture, children’s racial and ethnic background, and the culture of children’s community, and that tangible resources and support from professionals was critical for successful implementation. Barriers to SEL included limited time, lack of support, insufficient resources, devaluing the teaching of social and emotional competences, and programs that were not contextually relevant [41].

Studies have also examined how educators facilitate SEL through their everyday practice and interactions, without deliberate intervention (i.e., without utilising an explicit SEL program). Aubrey and Ward [42] used survey (n = 46) and follow-up interviews (n = 3) to explore the views of early years practitioners in the United Kingdom regarding early intervention for young children experiencing difficulties in personal, social, and emotional development (PSED). They identified a variety of strategies to strengthen PSED including modelling, establishing clear expectations, formally teaching social skills, dialogue and explanation, shared games and activities, and scaffolding. A similar mixed-methods approach was adopted by Hollingsworth and Winter [43] in the United States who explored preschool teachers’ beliefs relating to social–emotional competencies and the teacher practices that support these skills. In a study involving 32 Head Start and pre-kindergarten teachers, educators placed higher importance on social–emotional skills than early language, literacy, and math and employed a variety of responsive practices to promote prosocial skills, pretend play, and friendship formation. In contrast, Papadopoulou and colleagues explored the perceptions of 34 educators working in childcare centres in Greece. While educators acknowledged the importance of social and emotional competencies, they did not report consistent use of practices to promote these skills in the ECEC setting. The authors argued this may be influenced by a lack of formal pedagogical childcare curricula, guidelines or practices focused on social and emotional development, structural barriers, and a belief that social and emotional development is beyond their sphere of influence [44].

### 1.1. The Australian Context

SEL literature recognises that while children have common developmental needs, cultures differ in how competences are expressed, and approaches to aid SEL must empower children within their unique environment [45,46]. Similar to other countries, Australia has seen rapid interest and expansion in SEL across all levels of education, including ECEC. Australian ECEC providers operate under the National Quality Framework [47], incorporating national law and regulations, the National Quality Standard, the assessment and quality rating process, and learning frameworks including Being, Belonging, Becoming: Early Years Learning Framework 2009 (EYLF) [23]. This framework guides early childhood curriculum and pedagogy to address the developmental needs, interests, and experiences of each child, while taking into account individual differences. The EYLF describes the expected outcomes for all children who attend ECEC services, with a strong emphasis on social and emotional wellbeing, including that they have a strong sense of identity, are connected with and contribute to their world, have a strong sense of wellbeing, and are confident and involved learners and effective communicators. This Framework calls attention to child-centred and integrated teaching approaches to facilitate knowledge and skill acquisition, while recognising children learn at different rates, in different ways, and at different times. Further, it acknowledges educator practice and decision making is based on knowledge and skill, knowledge of children, families and communities, self-awareness of how their own beliefs and values impact children’s learning, personal styles, and past experiences. Underpinning the EYLF learning philosophy is contemporary knowledge of early social and emotional development, encouraging educators to consider children’s learning and development within daily practice, curriculum decisions, planning, and reflection. In Victoria, where the current study was conducted, the Victorian Early Years Learning and Development Framework, aligned to the pedagogy of the EYLF, also informs the practice of Victorian professionals working with young children [48].

### 1.2. The Present Study

National policy and frameworks set a clear expectation that Australian educators foster children’s social and emotional competencies. However, many educators have not received formal preservice or professional learning in SEL or strengthening children’s social and emotional capabilities through teacher–child interactions [41,49,50]. Furthermore, little is known about educators’ perceptions of how they encourage SEL within the early learning environment, and there lacks research investigating the barriers and enablers for SEL delivered as part of everyday ECEC practice.

To inform the development of an intervention to support educators to foster children’s social and emotional development in Australian early childhood settings, an understanding of educator knowledge and perceptions of their current practice was needed. Exploration of educators’ perspectives regarding children’s social and emotional development and the role they play could uncover important strengths and limitations in current practices, highlighting opportunities to support the sector to improve outcomes for young children.

The aim of this study, therefore, was to examine educators’ perspectives on children’s social and emotional development within the ECEC environment, the approaches educators use to encourage children’s social and emotional skills, the enablers and barriers for educators in fostering social and emotional skills within their classroom, and the supports that could strengthen practice across the sector. To address this topic in depth, a qualitative design with semi-structured interviews and focus group discussions was adopted.

## 2. Materials and Methods

### 2.1. Participants

A convenience sample of 30 professionals working within the ECEC sector were recruited to participate in this study [51]. Participants included early childhood educators working in kindergarten and long day-care rooms from four Melbourne-based ECEC providers (n = 20), 5 non-teaching staff who held a leadership or executive management position with oversight of ECEC service provision, 3 researchers with expertise in early child development within ECEC, and 2 staff from non-government agencies with knowledge or involvement in efforts to increase early social and emotional development. These non-teaching professionals were included to provide further insight and validation of educators’ perspectives. A list of potential participants was generated by the research team and invited to participate via email. The demographic characteristics of participants are provided in Table 1. The ECEC providers who participated in this study manage services within culturally diverse communities across the Greater Melbourne region.

### 2.2. Interviews and Focus Group Discussions

This study included both individual in-depth telephone interviews (n = 13) and in-person focus group discussions (n = 17). The inclusion of individual interviews and focus group discussions was informed by the availability and preference of participants. In-depth interviews were carried out with 13 professionals. Participants were provided a Plain Language Statement from the research team via email. Where informed, written consent was provided, an interview was conducted via phone by a member of the research team (CB). Each interview was audio-recorded. While recognising in-person interviews are often considered the gold standard [52], studies show telephone interviewing can offer an effective mechanism for data collection [53,54,55]. In one of the four participating ECEC centres, educators were invited to take part in a focus group discussion with their peers during a planned professional development session. All educators received a Plain Language Statement and a member of the research team described the project. Educators (17 in total) who provided informed, written consent were separated into three groups. A member of the research team facilitated each group discussion using the interview schedule, which was audio-recorded.

Interview questions were designed by the authors of this study. The topics in the interview guide (provided in Supplementary Materials) related to participants’ knowledge of children’s social and emotional development in early childhood, approaches utilised to support children to develop both socially and emotionally in ECEC settings, perceived barriers to SEL, and potential pathways to overcome these barriers. The structure of the discussions included an introductory statement on each topic (knowledge, approaches, barriers, and pathways), followed by a series of open-ended questions. Ethics approval to conduct the study was granted by the Deakin University Human Ethics Advisory Group.

### 2.3. Analysis

Inductive thematic analysis was used to identify the patterns and themes reported [56]. Our analyses were guided by the approach used by O’Connor, Nolan, Bergmeier, Williams-Smith, and Skouteris in their qualitative study investigating early childhood educator perspectives of supporting parent–child relationships and children’s social and emotional development [57]. Specifically, the five phases of inductive thematic analysis used were: (i) become familiar with the data; (ii) generate initial codes; (iii) search for themes; (iv) review themes; and (v) define and name themes [56]. Interviews and focus group discussions were transcribed verbatim. Two researchers (CB, AO) independently read, re-read, and coded 20% of transcripts to ensure the identification of consistent themes. Each transcript was read multiple times and initial codes identified. Similar initial codes were then grouped into more general themes, guided by the study aims. An Excel spreadsheet was used to assist with data management, coding, and analysis. Researchers compared and refined codes, themes, and subthemes, and any differences in independent coding were resolved by discussion. One author (CB) coded the remaining transcripts according to the coding structure. Members of the research team then reviewed and cross-checked codes, themes, and subthemes to ensure accurate coding of participant perspectives.

## 3. Results

Four core themes emerged from the analyses, described in detail below, relating to: (1) educator knowledge; (2) mobilising knowledge—social and emotional learning is embedded within interactions; (3) barriers—capacity and capability; and (4) strengthening educator skill—building knowledge through practical strategies. Quotes are verbatim comments from participants (E = Educator, individual interview, FG = Educator, focus group, and P = ECEC professional in a non-teaching role).

### 3.1. Educator Knowledge

Participants referred to a broad range of competencies to describe early social and emotional development. Social development was recognised through prosocial interactions and communication with peers, caregivers, and groups, ability to communicate own needs, and confidence to separate from parents or caregivers. Emotional skills included understanding, labelling, expressing, and regulating emotion, recognising emotion in others, and displaying empathy. Self-awareness and identity, confidence, responsible decision making, resilience, and coping were also identified. Professionals who were not employed directly within the classroom (i.e., ECEC leaders, researchers, and program managers) were more likely to describe social and emotional development as the demonstrated growth in capabilities against expected milestones or norms, compared with educators who described observed behaviours and reflected on their own encounters and experiences working with children. Only two participants discussed the relationship between social–emotional development and mental health; however, all recognised social–emotional functioning as one of the most important areas of focus for ECEC, and foundational for future development:

*Really for me social and emotional development is the most important thing to focus on for young children, I think all of the academic stuff will come if they have the right sort of social and emotional development [E08]*

When asked to describe the factors that influence children’s social and emotional skills, educators and other ECEC professionals referred to individual, interpersonal, and environmental aspects. At the individual level, child temperament, communication skills, stage of development, confidence, and resilience were identified. Most emphasised the importance of secure, consistent relationships with parents and caregivers, as well as other adults outside the family. In addition, organisational and environmental factors were acknowledged in many interviews as important determinants of social and emotional growth, including the home environment, general surroundings and experiences, cultural influences, high-quality early years learning programs, and exposure to environments that encourage exploration.

Participants pointed to various indicators of early social challenges. Behavioural problems including aggression, attention seeking, lack of initiative, and excessive physical expression were highlighted, as well as difficulty engaging in prosocial interaction and play, evident through high levels of conflict, lack of confidence, and social disconnection. A number of educators identified difficulty separating from parents, internalising behaviour, over-reliance on the educator, and speech and language delays as indicative of broader social difficulties. Similar themes were identified as markers of emotional problems. Several respondents also referred to dysregulated emotional responses as an important indicator of potential emotional challenges. ECEC professionals recognised the need to consider children’s behaviour in terms of what would be expected of a child that age, as well as the “normal” behaviour of the individual child.

Educators reported that they derived knowledge through structured learning experiences such as pre-service education, professional development, online training, and conferences, as well as educator-led learning through internet and print research, sourcing journal articles, and referring to theory. Many discussed drawing on their experiences in the early learning environment, working with children and families with diverse and individual needs, knowledge gained through the trial-and-error nature of the ECEC educator role, observation of their peers, insights from parents, knowledge gained from being a parent themselves, and interaction, discussion, and reflection with colleagues:

*It’s talking amongst our peers too, and observing, and learning as you go along with the children [FG5]*

Further, several highlighted the benefits of working closely with other professionals, including preschool field officers and early intervention services who can provide targeted and intensive strategies and assistance. One service offered educators monthly consultations with a clinical psychologist. During each session, an educator presented on a child exhibiting difficult or challenging behaviour. The group then worked together on strategies, and the psychologist offered input and advice in relation to mental health and possible influential family factors.

Educators were asked if they used a curriculum to support children’s social and emotional development. Responses were mixed. Several noted their practice was guided by the EYLF. Others did not use a specific curriculum but indicated practices to support social and emotional development were embedded within a shared philosophy and approach, experience and understanding of the dynamics of the children and group, planning for children’s learning, reflection, and professional development. A small number of respondents suggested they would welcome specific SEL curricula:

*I think a curriculum would be a great thing, because then you could be quite sure what all children are getting…. all the children would have the same foundation [E08]*

*I would say it’s possibly the forgotten area a little bit. I think there’s a big focus on literacy, a big focus on numeracy, STEM is the big buzz word at the moment, that everybody is focusing on, which is fantastic but as these things come into fashion, some of the other things fall off a bit and I think because social and emotional* [development] *is different, I don’t think there is a one size fits all…… just from an intentional teaching perspective, I think it’s something we could definitely do some more work on [E14]*

### 3.2. Mobilising Knowledge—SEL Is Embedded within Interactions

Participants described various approaches to support children’s social and emotional skills. These were embedded within relationships and interactions with children and families.

#### 3.2.1. Educator–Child Relationship

The educator–child relationship was widely acknowledged by educators and other professionals as critical to children’s development. Through a responsive and nurturing relationship, educators “tune into” children, gaining knowledge and understanding of each child’s individual needs. They can then identify concerns quickly and predict possible future behaviour. Staff from ECEC services (both educators and management) described this relationship in terms of attachment theory, emphasising that educators offer security by building trust and being emotionally available. One educational leader noted how this secure and safe base allows children to feel supported and build confidence as they experience “big feelings” and practise the skills to master them; the conversations children have with educators help them to make sense of what they are feeling.

*I think it’s just really being available to the children, so really being in tune with them, to their emotions and being emotionally available yourself so that you can respond to children appropriately, you know you can support them through managing their emotions appropriately [P03]*

#### 3.2.2. Supporting SEL through Everyday Interactions

Targeted strategies to support SEL were embedded within everyday experiences and interactions, during child-directed play and learning, guided play and learning, and adult-led learning. Educators discussed utilising a broad range of approaches tailored to children’s needs; however, there was variation in the breadth of strategies identified across interviews. As noted by an ECEC leader:

*Supporting children with social and emotional [development] shouldn’t sit separate to what we do every day with every child…it’s embedded in what we do for every child [P01]*

Educator role modelling was identified as especially beneficial for children experiencing behavioural difficulty. Educators discussed allowing children to observe and absorb appropriate behaviour without expectations and gradually participate at their own pace. Many educators also discussed assisting children in identifying and labelling their emotions, understanding there is nothing wrong with their feelings, and building strategies and skills to move forward. The following comment is representative of findings:

*We label the emotion, “I can see you’re really angry that your friend walked away from you, and I can see that made you upset, let’s try and solve the problem together” and getting in the moment rather than teaching on the mat [FG5]*

Tapping into teachable moments during child-led play was identified by several participants, who recognised the learning that occurs through responsive interactions and gentle conversations. Individual and small group discussions allow educators to work alongside children, talk about how they are feeling, and help them to identify the words to explain their emotions. Educators also described working with children through adult-led teaching, using social stories, structured activities, songs, books, games, puppets, and role play. As one educator noted, stories are an especially effective way to help children to identify with what might be happening around them, and how they and others might be feeling, thereby starting to develop empathy:

*Children are quite egocentric and our role is to get them to start thinking about how our behaviours and actions can affect other people as well [E14]*

#### 3.2.3. Physical Environment Supports SEL

The influence of the layout and organisation of the preschool classroom on children’s development was also highlighted. Educators noted visual aids (e.g., visual schedules and visual cards) are especially helpful for children with additional needs (requiring or able to benefit from specific considerations or adaptations). These can assist children in understanding and predicting what the day will look like, self-regulating, and controlling their emotions. Educators used the physical resources and materials within the room as a behaviour management technique—for example, redirecting children to activities or play stations which educators knew were enjoyable for that child, and setting up play spaces that responded to different sensory needs. The variety of play spaces and activities available encourage creativity, social interaction, and quiet time. As one educator explained:

*I think everything in a kindergarten setting is set up to help children socially and emotionally, from the types of play spaces that are provided, they’re active, there are quiet play spaces, there’s play spaces for one child, there’s places for four children, everything in the room is set up to help them engage socially in their environment and with the people around them [FG4]*

#### 3.2.4. Working with Caregivers

Participants consistently emphasised the importance of working in partnership with caregivers to support children’s social and emotional functioning. Parents were recognised as the most important influence on their child’s life, and therefore, any strategies implemented in the preschool classroom are unlikely to be effective without reinforcement and consistency at home. Educators built relationships with families, recognised the individual needs of the child and family, and worked with the family to identify and implement strategies. ECEC centres use a range of approaches to build this partnership, including intake interviews, questionnaires, meetings, making parents feel welcome when dropping off and picking up their child from the centre, online learning stories (written observations and photographs shared with caregivers via an online system), and home visits.

### 3.3. Barriers—Capacity and Capability

Several barriers to supporting social and emotional skills were identified: a lack of time to focus on SEL, group size, educator capability, confidence and training, high staff turnover, difficulty engaging with families, perceived lack of recognition of the educator’s role, and a lack of consistency across services. Most respondents identified that time constraints and high educator-to-child ratio (in Australia, centre-based ratio requirements are as follows: birth to 24 months: 1:4, between 24 and 36 months: 1:4 or 1:5 depending on the state, over 36 months and up to preschool age: 1:10 or 1:11 depending on the state) negatively influenced educators’ ability to embed social and emotional learning. Several emphasised children need one-on-one time to work through emotions and challenges at the time they experience them, but this is often not possible due to competing priorities. Challenges associated with a perceived increased proportion of children attending ECEC services with additional or undiagnosed additional needs were identified. These included ensuring the service caters to the needs of all children, the time required to complete documentation to access additional support, and the risk of overlooking the needs of both children experiencing social, emotional, and behavioural difficulties as well as typically developing preschoolers.

Educator capability to nurture social and emotional skills was identified as a potential barrier in several interviews, with respondents acknowledging capability is shaped by preservice education and professional development, experience in different settings, time in the sector, and motivation. Two participants in ECEC leadership positions suggested a lack of confidence and self-esteem can influence educator practice, with one noting educators often develop attachments with children and will instinctually know how to support social and emotional skills but lack confidence and belief in the importance of their role. Gaps in educators’ skillset were also identified by a small number of respondents, specifically working with children experiencing social and emotional difficulty, and engaging with parents whom the educator may perceive as difficult to communicate with. Educator motivation to up-skill and explore the resources available, and their ability to be mindful and reflective in prioritisation of activities, was also noted as a potential barrier (e.g., what is most important to do for the child). Participants working directly with children did not specifically discuss their capability to attend training. A number of respondents suggested tertiary training programs needed to place greater emphasis on social and emotional competencies, and evidence-based approaches to translate concepts to effective practice.

Notwithstanding the importance of working in partnership with families, engaging with caregivers regarding children’s social and emotional development was emphasised as a significant challenge, particularly due to parental expectations. For example, a number of educators noted a disconnect between some parents’ focus on literacy and numeracy, compared to social and emotional development, which was described as the critical developmental focus from the educator perspective. Educators can find it difficult to communicate the value of SEL to parents, while being respectful of their culture and beliefs. One educator described a lack of interaction with parents as a barrier to learning more about their child. In addition, the complexity of working with parents of children who may be experiencing difficulties was noted, particularly when the educator perceived parents were not ready or able to engage in a dialogue or accept that their child may be having difficulty.

*And a lot of families they don’t get it, that social and emotional is so important for school [it’s language and literacy] that if they’re not coping and they’re not managing at school just with their emotions and socially, they’re not going to be happy therefore they’re not going to learn and they don’t understand that [they don’t make that connection] [FG4]*

*Sometimes it’s hard to have that initial conversation with the parent, so you just have to know and understand what the family structure is about because you can’t sort of go in and go “your child’s got…”, you have be quite tactful [respectful]…One of the children in my room, it took me 12 months to have that conversation because it was never ever the right time and then one time it happened and I was there ready to go, you know [FG5]*

Educators and other professionals identified an extensive range of programs to support SEL within preschool. The KidsMatter Early Childhood Framework [58] had been utilised by several participants. Other programs which centres were currently or had previously implemented included Animal Fun [59], 1-2-3 Magic [60], Circle of Security [61], PALS Program [62], and Early ABLES [63]. However, some highlighted the volume of programs available and increasing expectations placed upon educators meant programmatic approaches were less likely to be embedded and sustained within ECEC services over time:

*…you know every couple of years there’s a revolution in childcare, everyone’s got a new idea and everyone’s got to implement the new idea and then you go back to 10 years ago, and go this worked then [FG4]*

Two educators noted the approach to implementation was ad hoc, with classrooms within the same centre independently selecting certain components or themes each year. Another respondent (researcher) discussed the lack of clarity regarding what a high-quality SEL pedagogy looks like, suggesting if you asked a group of educators to describe the key elements of SEL, the key capabilities, progression, and pedagogies that support learning, you would likely receive different responses:

*Educators can’t pick up a journal and see that example, a really well targeted description of what capabilities in SE look like, progression low to high, what effective pedagogy looks like, what effective measurement and clinical practice looks like [P10]*

### 3.4. Strengthening Educator Skill—Building Knowledge through Practical Strategies

Participants identified a need for support that responds to the unique context and requirements of ECEC centres, aligned with the National Quality Standard and EYLF, and not requiring additional time or resources to implement. That is, resources that are accessible, easy to use, and can be embedded into daily practice and routines, as reflected in the following comment:

*It needs to be something that’s easy to implement, that’s quickly accessible, that you can put into your program without having to think too much about it, so it naturally fits in, it links it with everything that’s already existing, it links with the NQS, it links with the EYLF, it links with all those things, but it’s not something else to learn, we don’t have to go oh my gosh, it’s another box we have to tick, it’s another thing we have to meet, it’s another criteria that has to be acknowledged in the program and then adding to it, it would be nice if it just fit nicely into the social and emotional area in everything [FG4]*

Up-skilling educators in practical strategies and techniques that foster SEL was suggested by several participants, who noted that tools need to respond to the different ways educators build knowledge, considering sensory learning styles and delivery modalities, for example:

*Educators always want practical stuff. Tell me how to do it, they like to have the information, but then give me the strategies, what do I have to do? So, practical stuff is really important, whether that’s conversation starters, actual sentences that you can use with children to support that, and I think video can be useful as well, just seeing how an educator does approach that kind of development in action is often, I think useful as well [E14]*

Coaching and mentoring were highlighted as effective in building capability within ECEC classrooms. Two ECEC providers had implemented a mentoring program to nurture and develop educator practice, with participants noting more was needed. Increased opportunity to reflect, collaborate, and share knowledge with team members was suggested. Greater focus on explicit social and emotional skill instruction in addition to warm and responsive play, approaches tailored to the developmental stage of the child, and greater emphasis on collecting and interpreting data relating to child progress were also raised as mechanisms to strengthen educator practice and child outcomes.

## 4. Discussion

The current study examined ECEC professionals’ understanding of early social and emotional development, the practices and approaches that encourage children’s social and emotional skills, the enablers and barriers for early childhood educators in fostering skill growth, and the additional resources and support that could assist in this respect. The findings suggest children’s social and emotional development is at the forefront of educator planning, practice and reflection, supporting findings from similar research with early childhood educators in the United Kingdom [42], United States [41,43,64] and Singapore [65]. Participants unanimously endorsed the role of early childhood educators in fostering children’s social and emotional skills, in partnership with families, as the building block for healthy learning and development.

Social–emotional competence is a multifaceted concept based on emotional, cognitive, and behavioural knowledge and skill [66]. The current study suggests early childhood educators recognise this breadth and complexity of social–emotional skills, and the linkages that exist between social and emotional competencies. For example, several participants discussed the importance of the secure attachment that can form between an educator and child, and how this encourages the child to feel safe and explore the social world and provides a model for social interactions on which to build future relationships.

Participants described utilising a broad range of approaches to support children’s social and emotional development, which could be grouped into four categories: (1) nurturing and responsive educator–child relationships; (2) supporting SEL through everyday interactions and practice; (3) utilising the physical environment to encourage SEL; and (4) working in partnership with caregivers. Similar groupings have been reported by other researchers who have classified SEL strategies in early childhood settings. For example, O’Conner and colleagues [67] identified three classroom factors (beyond using a SEL curriculum) associated with social and emotional skill development for children aged three to eight years: positive classroom climate (modifying the physical space and materials, classroom management strategies and routines, and a supportive and emotionally positive environment), instructional strategies (modelling, reacting to, and instructing about children’s emotional expression) and teacher’s own social and emotional competence (supported through direct training, reflective supervision and relationship building, and stress-reduction strategies).

Educators in this study appeared to value and utilise programs and interventions to support SEL at the class-wide level, yet perceived embedding SEL into everyday interactions as the most effective way to foster children’s strengths and meet the unique needs of each child in the moment. Hollingsworth and Winter [43] similarly reported teachers foster prosocial skills by setting the tone of the social environment and responding to situations as they arose. This points to the potential utility of both universal class-wide SEL intervention in addition to fostering every child’s SEL through tailored and responsive supports embedded within interactions. Knowledge and insight from families was also recognised as vital for children’s SEL. Educators work with caregivers to understand the unique needs and context for each child, identify proactive, preventative, and early intervention strategies, and scaffold learning across home and service settings. In early childhood, family–child relationships are the primary source of learning experiences. SEL activities in the preschool environment can be reinforced and enhanced by enlisting families as partners in the overall SEL approach [28,68].

The findings of this research, however, indicated inconsistency across ECEC providers in the variety and type of strategies educators use to support social and emotional skill growth. There may be a lack of guidance on translating the EYLF into practice with regard to preschoolers’ social and emotional development. As a result, children’s exposure to high-quality interactions, strategies, and techniques that facilitate SEL is influenced by the knowledge, skill, and confidence of educators, and the culture, leadership, philosophy, and structural quality of each service, including educator-to-child ratio, space, resources, staff qualifications, programs, and curricula. This aligns with a recent exploration of Australian early childhood educators’ perceptions of the EYLF. This research reported educators are engaged with the content but seeking ways to transform the professional concepts embedded in the EYLF into their practice [69].

Teaching in early childhood is often characterised by continuous analyses of children’s understanding, and decisions about curriculum and pedagogy. This highlights the importance of understanding the knowledge educators draw on in their decision-making processes [70]. Regarding how educators understood and conceptualised social and emotional development, and how they perceived they supported children’s social and emotional skills, participants in this study appeared to draw on both explicit and tacit knowledge. Explicit knowledge refers to objective and rational knowledge that can be easily transferred through words and sentences [71]. It is knowledge that educators are consciously aware that they are using and that can be documented and communicated—for example, the EYLF, manualised SEL programs, and the step-by-step description of strategies where educators could clearly articulate why they were using the approach and the desired outcome for children. Tacit knowledge, by contrast, is personal and subjective, relating to the mental models, values, beliefs, perceptions, insights, and assumptions that educators form when working with children and families over time [72,73]. This knowledge may be more difficult to transfer to others. Across several interviews, educators suggested their practice was based in their knowledge and relationships with children, but they found it challenging to describe the specific strategies and techniques applied, which may reflect their tacit knowledge base.

Making educator knowledge explicit is critical to support teacher learning [74]; however, research indicates much teacher knowledge is implicit and not articulated [75]. Reliance on tacit knowledge also limits the opportunity to replicate high-quality, evidence-based practice across settings. The findings of this study highlight that although social–emotional development is a priority for early years professionals, there is inconsistency in training and application of support to enable this to occur. ECEC professionals seek practical strategies that will support them to strengthen children’s social and emotional skills through their everyday interactions and practice. Building upon educators’ practical (tacit) knowledge through the provision of explicit, documented techniques tailored to the breadth of strengths and needs within the room could allow educators to integrate formal learning with personal experience. More could also be done to assist educators in connecting with families to foster social–emotional development within the home environment.

## 5. Study Limitations

There are several limitations to this study. First, it relied on a convenience sample. While the authors invited educators from several different ECEC providers and included non-teaching ECEC professionals to validate the findings, these cannot be generalised, as is the nature of qualitative research. This approach was, however, appropriate to address the research aims, representing the perspectives of the participants, and feasible within the time and resourcing constraints of the study. Second, the research methodology included both in-depth interviews and focus group discussions. Recognising it would have been preferrable to individually interview all participants, this format was most feasible for participants, and a pragmatic decision to integrate both methods was made. Furthermore, this research focused on educators’ perceptions of supporting children’s social and emotional development in the ECEC environment. This is important because educators’ perceptions and judgments affect teaching practice. However, it is not possible to draw conclusions regarding their actual practices and behaviour. It would be beneficial to conduct observations of educators in the early learning classroom to better understand what teachers do in practice. Finally, while this study provides insights on the perspectives of Australian ECEC professionals, we did not explicitly explore how participants’ beliefs and practices were informed by either their own cultural background, or the cultural background of children in their care. While beyond the scope of the current study, this would be valuable to consider in future research.

## 6. Conclusions

This study adds to the literature through its focus on how the Australian ECEC sector approaches SEL in preschool classrooms. Aligned to the EYLF, ECEC professionals uniformly conceptualised children’s social and emotional skills as critical to ongoing development and a primary focus for the sector. However, findings suggest the breadth of strategies and techniques to support this development vary across organisations, influenced by a range of factors including structural quality, educator knowledge, skill and confidence, and qualifications and experience. Educators acknowledge trial and error is necessary in early years settings, and an approach that works for one child may not have the same impact or benefit for the next. Attention towards ensuring all children receive the type of interactions that will support positive social and emotional outcomes is warranted. Strengthening knowledge through a variety of explicit and practical strategies that can be embedded into daily practice was recommended by participants. These findings will inform the development of a pedagogical intervention to promote SEL and positive mental health in preschool classrooms and may offer valuable insights to support the pedagogical practices championed in Australian early learning policy.

## Figures and Tables

**Table 1:** Demographic characteristics of the participants.

	N	%
All Participants		
Gender		
Female	29	96.7
Male	1	3.3
Role		
Early Years Educator	20	66.7
ECEC Manager, Executive or Advisor	5	16.7
Academic Researcher	3	10.0
Program Manager	2	6.7
Early Years Educators		
Age (years)		
25–29	1	5.0
30–39	6	30.0
40–49	7	35.0
50–59	3	15.0
60+	3	15.0
Qualification		
Masters	1	5.0
Bachelor	4	20.0
Graduate Diploma	1	5.0
Advanced Diploma	1	5.0
Diploma	10	50.0
Certificate III	3	15.0
Experience in ECEC Sector (years)		
0–5	1	5.0
6–10	7	35.0
11–20	6	30.0
21–30	3	15.0
31–35	3	15.0
Employment		
Full Time	9	45.0
Part Time	11	55.0

## Data Availability

Data sharing is not applicable to this article.

## Supplementary Materials

The following are available online at https://www.mdpi.com/1660-4601/18/4/1530/s1, Table S1: Interview Guide.
