# Supplementary material for: “It’s Embedded in What We Do for Every Child”: A Qualitative Exploration of Early Childhood Educators’ Perspectives on Supporting Children’s Social and Emotional Learning
Source: Int J Environ Res Public Health. 2021 Feb 5;18(4):1530. doi: 10.3390/ijerph18041530 (PMC7914528; doi:10.3390/ijerph18041530)
Supplement: Supplementary file 1 [file ijerph-18-01530-s001.pdf]

Interview Schedule (semi-structured interview/focus group)

Social and emotional learning in early childhood education and care settings

Questions for Educators/ECEC Managers *(possible prompts in italics)*

General

- What is your age? (provide age ranges: 18-24, 25-29, 30-34, 35-39, 40-44, 45-49, 50-54, 55-59, 60+)
- What is your current position? (type of service, age of children under care, full time/part time/casual).
- How long have you been in your current position?
- How long have you been working as an early childhood educator/manager?
- Can you please tell me your highest level of education?

Knowledge of social and emotional development

- Can you please describe what you think social and emotional development is for children?
- What factors affect a child's social and emotional development?
- How might you recognise children who are having difficulty socially?
- How might you recognise children who are having difficulty emotionally?

Current approaches

- Can you tell me about the strategies and approaches you (educator)/your center (managers) currently use to help children develop socially?
- Can you tell me about the strategies and approaches you (educator)/your center (managers) currently use to help children develop emotionally?
    - *E.g. PD for educators, educator-child relationships, modeling, classroom/group activities, story-telling, play*
- What resources, tools or supports help you support young children's social and emotional development? These may be informal or formal.
    - *E.g. VEYLDF resources, training/PD, books/online resources etc.*
- Do you speak/engage with parents regarding children's social and emotional development?
    - *How do you go about this?*
- What are the barriers for you (educator)/your centre (managers) in supporting social and emotional development in children?
    - *E.g. time, resources, knowledge and skill, confidence.*

Part 3: Potential pathways

- What might help you overcome these barriers?
    - *What tools, resources or supports do you think would work in your room?*
